# Automated radiosynthesis and biodistribution of [^18^F]fluoroflutamide

**DOI:** 10.1186/s41181-026-00453-z

**Published:** 2026-07-23

**Authors:** Gina Kaup, Jason Anthony Witek, Shelbie Jaylene Cingoranelli, Jenelle Stauff, David Raffel, Allen Frederick Brooks, Peter James Henry Scott, Jay Samuel Wright

**Affiliations:** 1 Division of Nuclear Medicine, Department of Radiology, University of Michigan Medical School, Ann Arbor, MI 48109 USA; 2 The Interdepartmental Program in Medicinal Chemistry, University of Michigan, Ann Arbor, MI 48109 USA; 3 Department of Radiology, Perelman School of Medicine, University of Pennsylvania, Philadelphia, PA 19104 USA

**Keywords:** Androgen receptor, Prostate cancer, Metastatic castration-resistant prostate cancer, Radiofluorination, Positron emission tomography

## Abstract

**Background:**

The androgen receptor (AR) is upregulated in multiple cancers, such as salivary gland cancer, glioblastoma, triple-negative breast cancer, and, most commonly, prostate cancer. Imaging expression of the AR using a radiolabeled AR antagonist would enable non-invasive clinical staging and assessment of tumor heterogeneity via positron emission tomography (PET), complementing the findings of a [^68^Ga]PSMA-11 scan. Previous efforts described the radiolabeling of enzalutamide with fluorine-18 but were limited by low molar activity. In this study, we showcase an efficient, reproducible automated synthesis of [^18^F]fluoroflutamide via zinc-mediated radiofluorination and assess its biodistribution in preclinical models of prostate cancer.

**Results:**

From 1.87 Ci (69.19 GBq) of [^18^F]fluoride, [^18^F]fluoroflutamide was isolated with a non-decay-corrected radiochemical yield of 3.46 ± 1.30% (*n* = 4) and a radiochemical purity of > 99% in an average 79 min from end-of-beam using a bromoamide precursor. The average A_m_ was 1.57 Ci/µmol (or 58.23 GBq/µmol) at the end of synthesis, and the radiochemical purity after 4 h was > 97%, indicating negligible radiolysis. A biodistribution and imaging study was performed using nude athymic mice with 22Rv1 human prostate cancer xenografts. From the results of this study, we observed a ‘mass effect’ in which carrier-added mice displayed an increased uptake of the tracer across most organs.

**Conclusions:**

Herein, we report the optimized automated radiosynthesis of [^18^F]fluoroflutamide with high purity and sufficient yield for preclinical evaluation of AR-targeted imaging in mouse models of prostate cancer. The lack of uptake in androgen receptor-rich tissues and xenografts suggests impaired in vivo target engagement. While α-tertiary fluorination may attenuate CYP1A2-mediated oxidation to the higher-affinity 2-hydroxyflutamide metabolite, the observed signal is also consistent with reduced intrinsic AR affinity and/or altered pharmacokinetics of the fluorinated analog.

**Supplementary Information:**

The online version contains supplementary material available at https://doi.org/10.1186/s41181-026-00453-z.

## Background

The androgen receptor (AR), when bound by testosterone or dihydrotestosterone, functions as a transcription factor that initiates expression of genes required for normal sexual development and androgen-dependent physiology. Multiple studies have concluded that the AR is upregulated in certain diseases such as glioblastoma, (Zalcman et al. 2018) salivary gland cancer, (Fan et al. 2000; Xu et al. 2019) and triple-negative breast cancer (Rampurwala et al. 2016). However, interest in the AR has been directed mainly toward prostate cancer (PC) applications, as activation of the receptor leads to cancer progression. AR antagonists are first-line treatments for patients with PC, but therapeutic resistance typically accompanies disease progression to more lethal stages, such as castration-resistant prostate cancer (CRPC). Specific CRPC mutants are paradoxically activated by AR antagonists, leading to transcription of AR target genes that drive cancer growth. After discontinuing treatment with AR therapeutics, this subset of patients shows clinical improvement, which has been characterized as “anti-androgen withdrawal syndrome”(Watson et al. 2015). Additionally, an aggressive form of metastatic-CRPC that can arise from lineage plasticity in response to AR antagonists is neuroendocrine prostate cancer (NEPC), which makes up 10–17% of advanced/treatment-resistant PCs. Although rare, NEPC can also develop *de novo*, representing less than 2% of all PC cases (Wishahi 2024). 

To understand the AR expression pattern for disease staging and treatment planning, invasive tumor biopsies are performed. Heterogeneity, however, can mislead conclusions about the status of all tumors (Jamroze et al. 2021). Positron emission tomography (PET) imaging is a noninvasive alternative for staging PC progression and for determining the expression of prostate-specific membrane antigen (PSMA), which is located on the cell surface of prostate metastases. Currently, [^68^Ga]PSMA-11 is widely used for PET imaging of PC. As CRPC tumors acquire AR independence, PSMA is increasingly upregulated, thereby facilitating [^68^Ga]PSMA-11 PET imaging (Rosar et al. 2022). However, as neuroendocrine differentiation occurs, tumors largely lose PSMA expression, diminishing the imaging utility of [^68^Ga]PSMA-11 and eliminating the option for targeted radionuclide therapy (TRT) with [^177^Lu]PSMA-617 (Bakht et al. 2018). To complement PSMA imaging, targeting the AR with a PET agent would provide insight into the mechanisms underlying lineage plasticity to NEPC and androgen independence in CRPC. Furthermore, a fluorine-18 AR imaging agent could guide the development of alternative TRT platforms for prostate cancer based on matched radiohalide congeners (e.g., ^131^I, ^77^Br, ^211^At).

**Fig. 1:**
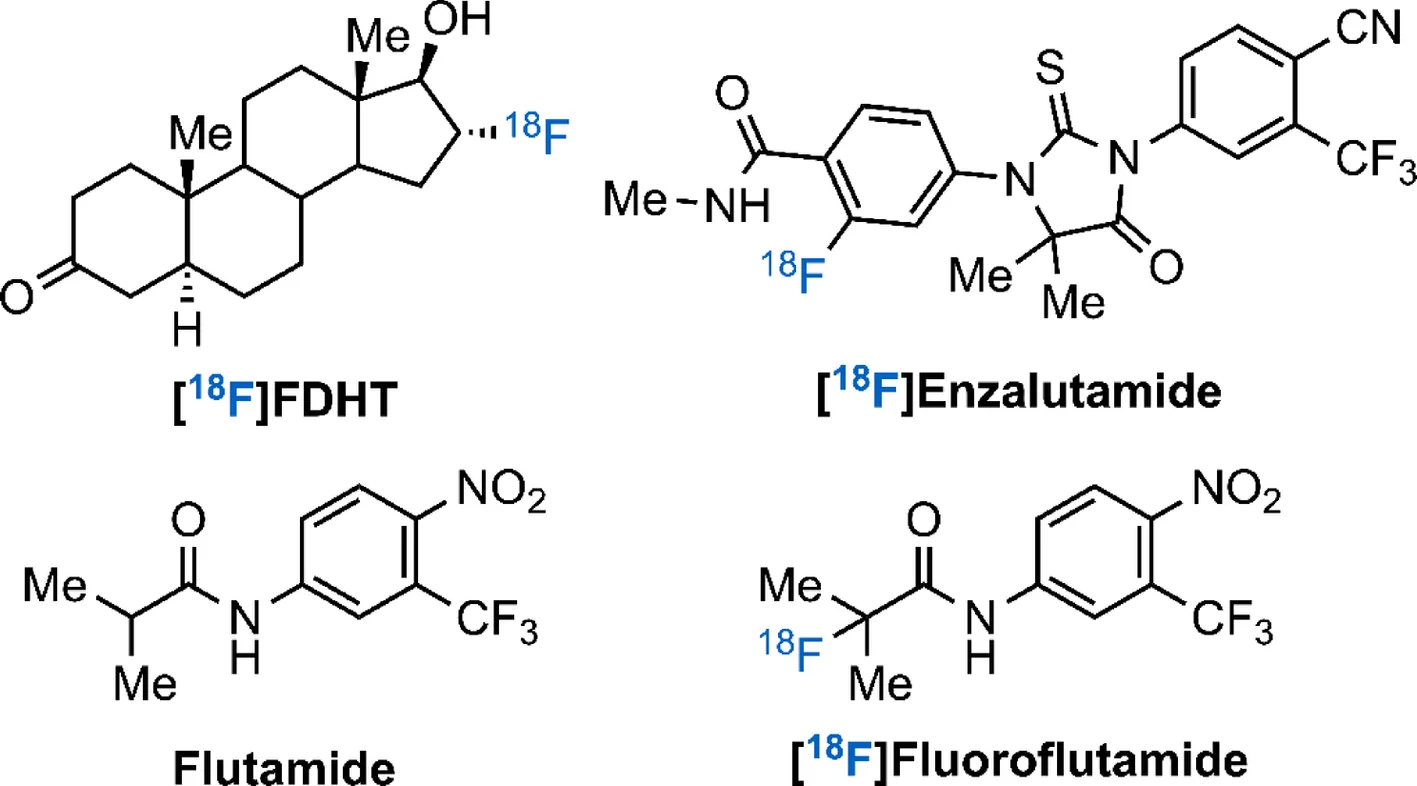
Structures of [^18^F]FDHT, [^18^F]enzalutamide, flutamide, and [^18^F]fluoroflutamide (**1**).

The testosterone derivative, 16β-^18^F-fluoro-5α-dihydrotestosterone ([^18^F]FDHT, Fig. 1), is an established AR agonist radioligand that has been investigated in clinical trials for PC imaging (Larson et al. 2004; Zanzonico et al. 2004; Dehdashti et al. 2005; Fox et al. 2011; Vargas et al. 2018; Fox et al. 2018; Jalali et al. 2023). The uptake of [^18^F]FDHT and [^18^F]fluorodeoxyglucose ([^18^F]FDG) in patients with PC was compared in a 2018 study along with their prognostic capabilities (Fox et al. 2018). The researchers determined three tumor subtypes: AR-positive and glycolytic-positive, AR-positive and glycolytic-negative, or AR-negative and glycolytic-positive. The last displayed the worst prognosis, consistent with low or absent AR expression, which correlates with more aggressive phenotypes. More recently, the uptake of [^18^F]FDHT and [^68^Ga]PSMA-11 was compared alongside immunohistochemistry of AR and PSMA expression in PC lesions (Jalali et al. 2023). The results showed a strong correlation between [^18^F]FDHT-positive lesions and AR expression, unlike that observed with [^68^Ga]PSMA-11 and PSMA expression. Heterogeneous AR expression was observed in 3 of 10 subjects, and an inverse relationship between AR and PSMA expression was observed across the cohort. These two studies further emphasize the promise and potential of an AR-targeted PET scan.

Despite its established binding, [^18^F]FDHT has shown inconsistent uptake in lesions and can obfuscate metastatic disease near the bladder. This is attributed to its renal clearance, which results in lower image resolution. The encouraging results from these studies have led researchers to identify other AR-targeted PET agents with more favorable imaging properties and biliary excretion. In 2021, the second-generation AR antagonist, enzalutamide (Fig. 1), was radiolabeled with fluorine-18 and used to image LNCaP (AR^+^ human prostate cancer) xenografts, and the results were compared with those of [^18^F]FDHT (Antunes et al. 2021). [^18^F]Enzalutamide displayed higher uptake in AR-expressing organs, lower background uptake, and higher plasma stability. Although its properties offer potential advantages, the low molar activity (0.68 Ci/µmol or 25 GBq/µmol) compromised image resolution. Here, we develop a high molar activity automated radiosynthesis to label an analog of the first-generation AR antagonist, flutamide (Fig. 1). Analogs of flutamide have been previously labeled with PET (^11^C, ^18^F, ^76^Br) (Liu et al. 2018; Jacobson et al. 2006; Parent et al. 2006) and SPECT (^99m^Tc) (Dallagi et al. 2010; Cardoso et al. 2023) radioisotopes, with structural modifications to enhance labeling compatibility. In this study, we use our recently reported zinc-mediated radiofluorination to label flutamide with fluorine-18 at a new position and evaluate its uptake in AR-rich tissue in vivo.

## Methods

### General considerations

Supplementary data, including figures S1–S24, can be found in the Supporting Information. All experimental details for syntheses and radiosyntheses, including radio-high-performance liquid chromatography (radio-HPLC) traces and nuclear magnetic resonance (NMR) spectra, are provided in the Supporting Information. Reagents and solvents were commercially available unless otherwise stated. Tetramethylammonium triflate (TMAOTf) was synthesized and characterized, with data consistent with previous reports (Wright et al. 2024; Sagl and Martin 1988; Toro et al. 2014). Compound **2** and the fluorine-19 standard of **1** were prepared according to the procedures described by Mizuta, and the spectra obtained matched those reported (Mizuta et al. 2021). 

### Radiosynthesis of [^18^F]fluoroflutamide

**Fig. 2:**
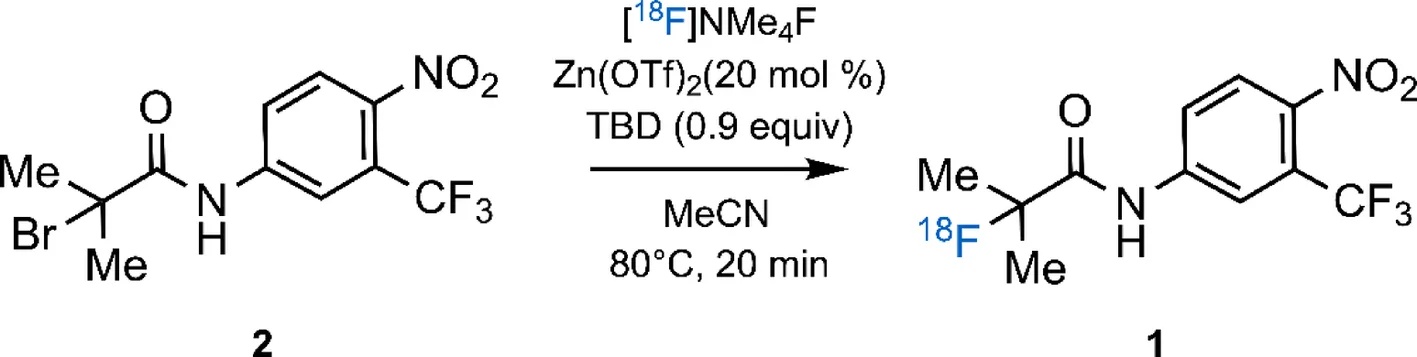
Conditions for the synthesis of [^18^F]fluoroflutamide (1) using zinc-mediated radiofluorination.

[^18^F]Fluoride was produced via (Liu et al. 2018) O(p, n)^18^F nuclear reaction (30 min beam at 55 µA, 1.87 Ci, 69.19 GBq) with a General Electric (GE) PETtrace 800 cyclotron and delivered to a GE TRACERlab FX N Pro synthesis module. The [^18^F]fluoride was trapped on a Waters Sep-Pak Plus Light QMA solid phase extraction (SPE) cartridge pretreated with 10 mL of ethanol (EtOH), followed by 10 mL of 1M potassium triflate (KOTf), and then 10 mL of Milli-Q H_2_O. The activity was eluted off the cartridge via ion-exchange with TMAOTf (5.9 mg, 26.5 µmol) in 500 µL of H_2_O to afford the nucleophile, [^18^F]TMAF, as described in our previous method (Wright et al. 2024). Subsequent addition of Kryptofix (K_2.2.2_) (0.14 mg, 0.36 µmol) in 1 mL of acetonitrile (MeCN), followed by azeotropic drying, produced anhydrous [^18^F]TMAF. In this context, the role of K_2.2.2_ is to suppress the production of [^18^F]HF by behaving as a non-volatile organic base and to capture any residual [^18^F]KF introduced by the KOTf wash. The precursor (**2**, 1.78 mg, 5.00 µmol) dissolved in 500 µL of MeCN was added to the reactor, followed by the addition of zinc triflate (Zn(OTf)_2_) (0.36 mg, 1.00 µmol) and triazabicyclodecene (TBD, 0.63 mg, 4.50 µmol) dissolved in 500 µL of MeCN (Fig. 2). The reaction was allowed to stir for 20 min at 80 °C, quenched (3.5 mL of HPLC buffer), and purified via semi-preparative HPLC (column: Phenomenex Luna C18(2) 10 μm 250 × 10 mm; mobile phase: 50% EtOH, 10mM NH_4_OAc, 30mM AcOH, 1mM ascorbic acid; measured pH = 4.8, flow rate: 4 mL/min). The product peak (t_R_ ~ 15 min) was collected for 1 min (4 mL) and diluted with 60 mL of Milli-Q H_2_O containing 0.1 mL of ascorbic acid injectable solution (500 mg/mL). The resulting solution was passed over a Waters Oasis^®^ HLB resin (30 mg/1 cc) and rinsed with 10 mL of sterile H_2_O. The product was eluted from the resin with 500 µL of USP ethanol, formulated with USP saline and 0.1 mL of ascorbic acid injectable solution (10 mL dose; 94% saline, 5% EtOH), and sterile-filtered through a 0.22 μm filter into a dose vial. Identity and purity were confirmed via analytical radio-HPLC.

### HPLC analysis

**Fig. 3:**
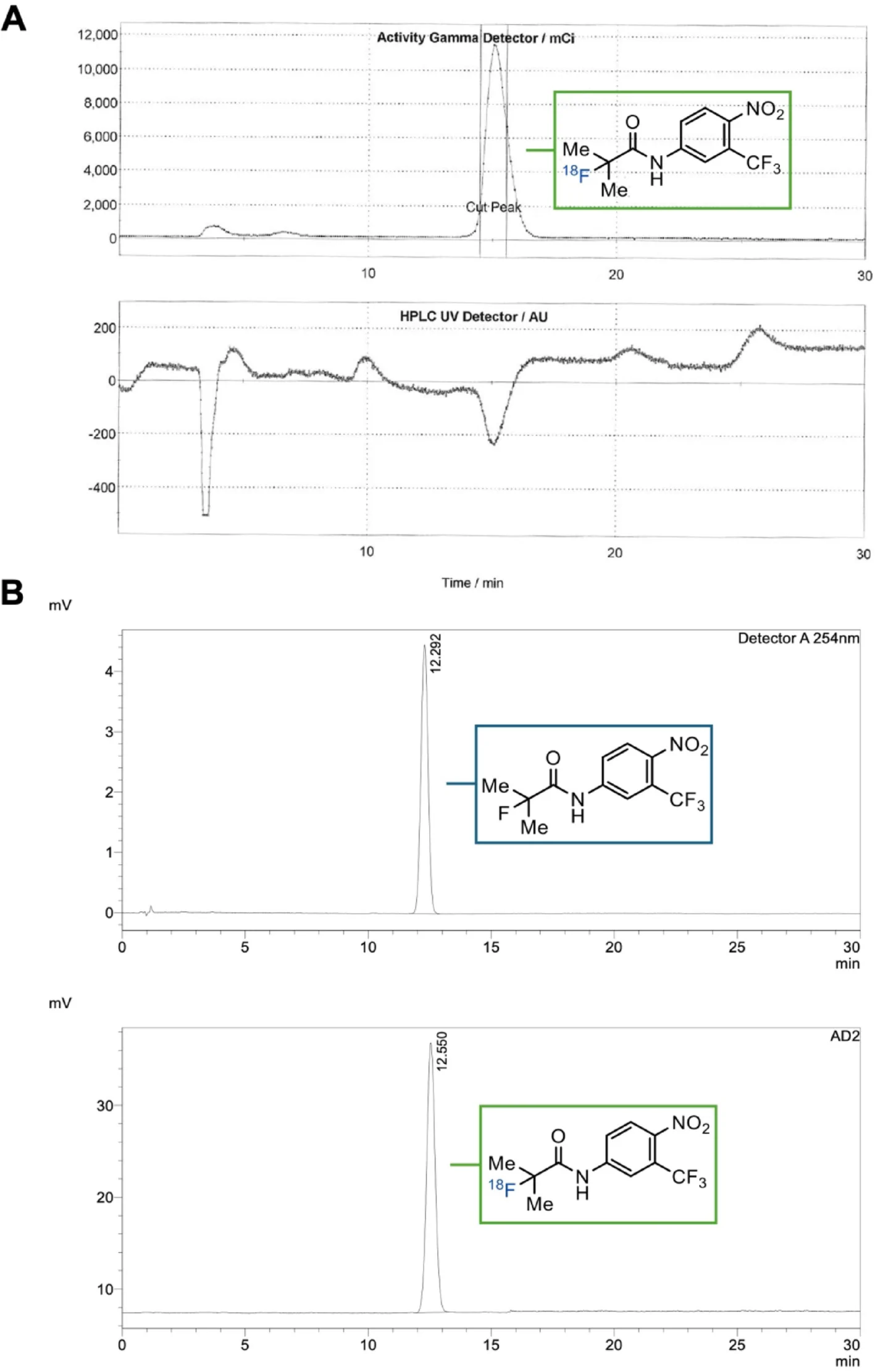
**A** Representative semi-preparative HPLC trace for the purification of [^18^F]fluoroflutamide (t_R_= 15 min). Column: Phenomenex Luna C18(2) 10 μm 250 × 10 mm; mobile phase: 50% EtOH, 10 mM NH_4_OAc, 30 mM AcOH, 1 mM ascorbic acid; pH = 4.8, flow rate: 4 mL/min. **B** Analytical UV HPLC trace of the fluorine-19 standard (upper) and gamma trace of **1** (lower). Column: Phenomenex Luna C18(2) 5 μm 150 × 4.6 mm; mobile phase: 40% MeCN, 10 mM NH_4_OAc, 30 mM AcOH; pH = ca. 4.3; flow rate: 2 mL/min.

The chemical and radiochemical purity of [^18^F]fluoroflutamide was analyzed using a Shimadzu LC2010 HPLC equipped with a Bioscan/Eckert and Ziegler radioactivity detector and an ultraviolet (UV) detector (Phenomenex Luna C18(2) 5 μm 150 × 4.6 mm; mobile phase: 40% MeCN, 10mM NH_4_OAc, 30mM AcOH; pH = ca. 4.3, flow rate: 2 mL/min, t_R_ ~ 12.5 min). A representative HPLC trace is shown in Fig. 3. Additional details are provided in the Supporting Information.

### Animal biodistribution and imaging studies

Biodistribution and imaging studies were performed in accordance with protocols approved by the University of Michigan Institutional Animal Care & Use Committee (IACUC). Male nude athymic mice bearing 22Rv1 human prostate carcinoma xenografts (5 × 10^6^ Cells in PBS/Matrigel, 1:1) were used for all studies. Tumors were allowed to grow for approximately 2 weeks until palpable prior to imaging. Mice were anesthetized with isoflurane (5% induction, 1–2% maintenance) and administered [¹⁸F]fluoroflutamide via tail vein injection (average activity: 125.33 ± 40.08 µCi, 4.64 ± 1.48 MBq). Animals were divided into three groups (*n* = 3 per group): tracer only (baseline), tracer co-administered with apalutamide (2 mg/kg), and tracer co-administered with ¹⁹F-fluoroflutamide (2 mg/kg) to assess AR-mediated binding and nonspecific uptake.

Since apalutamide and fluoroflutamide exhibit limited aqueous solubility and are typically administered via non-intravenous routes, formulation conditions enabling co-administration with the PET tracer were first established. Carrier-added doses were estimated based on reported oral bioavailability and evaluated across multiple solvent systems (Sulochana et al. 2018; Posti et al. 2000; Schulz et al. 1988; Vries et al. 2019; Clegg et al. 2012). Both compounds exhibited poor solubility in saline containing 0–10% ethanol; however, complete dissolution at 2 mg/kg in 50 µL injection volumes was achieved using a formulation comprising polysorbate 80 (Tween^®^ 80), ethanol, and saline. Formulations were agitated, sterile-filtered (0.22 μm), and analyzed by HPLC to confirm solution-phase stability prior to in vivo use (Kerwin 2008). 

For imaging, one mouse from each group underwent dynamic PET acquisition from 0 to 60 min post-injection (21 frames), followed by a static scan at 2 h post-injection using a small-animal PET system (MR Solutions). Images were corrected for decay, dead time, and random coincidences, and reconstructed using iterative ordered subset expectation maximization with maximum a posteriori (OSEM-MAP). Regions of interest were defined using Imalytics Preclinical software, and time-activity data were quantified as standardized uptake values (SUVs). For biodistribution, mice were euthanized at 2 h post-injection, organs were harvested, weighed, and counted for radioactivity using a gamma counter. Data were expressed as percentage injected dose per gram of tissue (%ID/g). Statistical comparisons were performed using one-way ANOVA and independent t-tests (GraphPad Prism).

## Results and discussion

Non-invasive imaging of AR expression would be beneficial for multiple cancers, including late-stage and aggressive NEPC. Increased interest in PC imaging agents has coincided with the recent FDA approvals of [^177^Lu]PSMA-617 (Pluvicto) and kit-based [^68^Ga]PSMA-11 formulations (Locametz/Illuccix). The first-line therapies for PC include AR antagonists such as enzalutamide, apalutamide, and darolutamide, which compete with androgens for AR binding. Through resistance pathways, AR dependency is eventually lost, coinciding with a waning of treatment response. In some instances, therapy continuation can facilitate disease progression, so PET imaging studies that accurately stage this transition would be invaluable for reassessing treatment decisions. Biopsies are currently used to evaluate AR expression, an invasive procedure that does not always provide a comprehensive understanding of target expression in each tumor. Therefore, by radiolabeling one of these AR antagonists with fluorine-18, we aimed to assess the ability of this agent to non-invasively measure AR expression through PET imaging.

Flutamide is a first-generation AR antagonist containing an α-2°-amide with a known active metabolite formed via α-hydroxylation. Recent methodology from our lab describes a wide array of α-amide substrates that can be labeled via zinc-mediated radiofluorination. In the case of 2-hydroxyflutamide, the active metabolite generated via CYP1A2, fluorine can also act as an isostere for the hydroxyl group due to its weak hydrogen-bond-accepting properties and high electronegativity (Inoue et al. 2020). Using this chemistry, we developed an automated procedure for radiolabeling flutamide for in vivo assessment. Our initial conditions for radiolabeling used dimethyl sulfoxide (DMSO) as the reaction solvent to develop a Class III solvent procedure. However, molar activity (A_m_) at the end of synthesis obtained with this method was lower than that obtained for [^18^F]enzalutamide (0.46 Ci/µmol or 16.95 GBq/µmol vs. ca. 1 Ci/µmol or 37 GBq/µmol), which would likely compromise image resolution. Gratifyingly, switching the solvent to MeCN increased the molar activity to 2.21 Ci/µmol (81.86 GBq/µmol).^23^ Control experiments in DMSO showed no radionuclide incorporation by TLC when the bromoamide precursor was substituted for flutamide under the optimized conditions, ruling out fluorodenitration and isotopic dilution at the trifluoromethyl group as contributors to molar activity reduction. Ongoing studies are investigating the substrate-specific formation of side products with chromatographic behavior similar to that of **1**, which could reduce effective molar activity in DMSO (Adams et al. 1999). 

With the optimized reaction conditions in hand, we pursued a fully automated synthesis, purification, and dose formulation of [^18^F]fluoroflutamide in MeCN on a GE TRACERlab FX N Pro for preclinical studies. [^18^F]Fluoroflutamide was isolated with an average non-decay-corrected radiochemical yield (RCY) of 3.46 ± 1.30% (*n* = 4) in > 99% radiochemical purity (RCP, Fig. 3B). The activity yields (64.55 ± 24.28 mCi) were sufficient for planned animal studies with an average synthesis time of 79 min. Additionally, the doses had an average molar activity of 1.57 Ci/µmol (or 58.23 GBq/µmol, see S7 and S22 for the standard curves) at the end of synthesis. Initial optimization of the automated method without ascorbic acid indicated radioimpurities originating from radiolysis 2 h after the end of synthesis in a 5% ethanol, 95% saline solution. By adding ascorbic acid to the dilution flask, the HPLC buffer, and the final product vial in addition to the ethanol, we observed negligible radiolysis of the final dose formulation for up to 4 h post-end-of-synthesis (99.54% RCP at 1 h; 97.81% RCP at 4 h).(Scott et al. 2009) See the Supporting Information for more details.

**Fig. 4:**
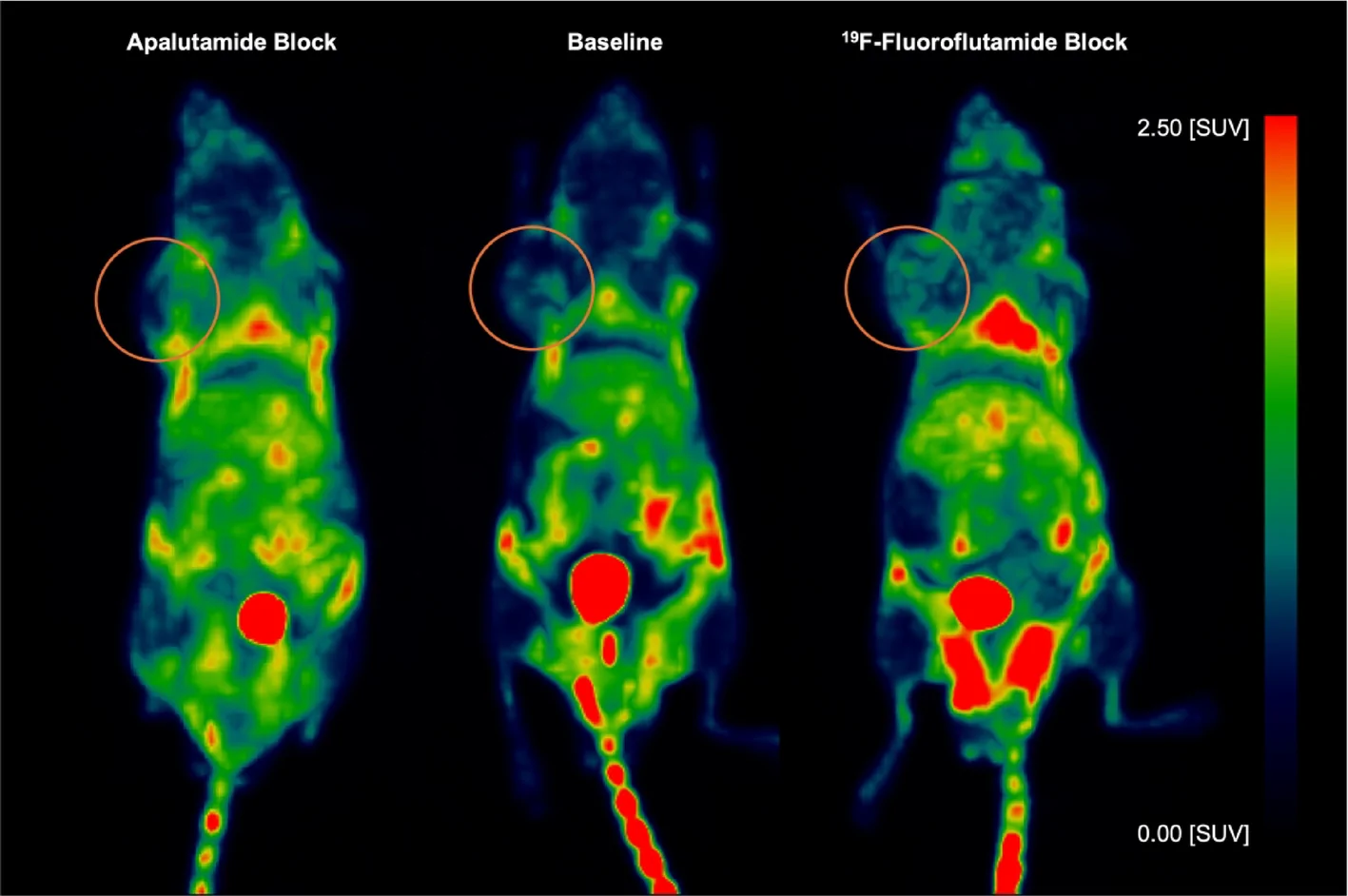
Maximum intensity projection of static PET images at 2 h post-injection of nude athymic mice with 22Rv1 xenografts (orange circle represents tumor). Standardized uptake values are represented as SUV_max_.

Following the development of a consistent, automated protocol, we performed biodistribution and imaging studies with [^18^F]fluoroflutamide in nude athymic mice bearing 22Rv1 tumors. Nine male mice were administered [^18^F]fluoroflutamide, and biodistribution studies were conducted 2 h post-injection. Of the nine mice, two groups of three were dosed with cold fluoroflutamide or apalutamide to evaluate specific binding. Dynamic PET imaging studies of one baseline mouse and the two blocking studies were completed from 0 to 60 min post-injection, with an additional 10 min static PET image recorded at 2 h. The maximum-intensity projections from these scans are shown in Fig. 4.

**Fig. 5:**
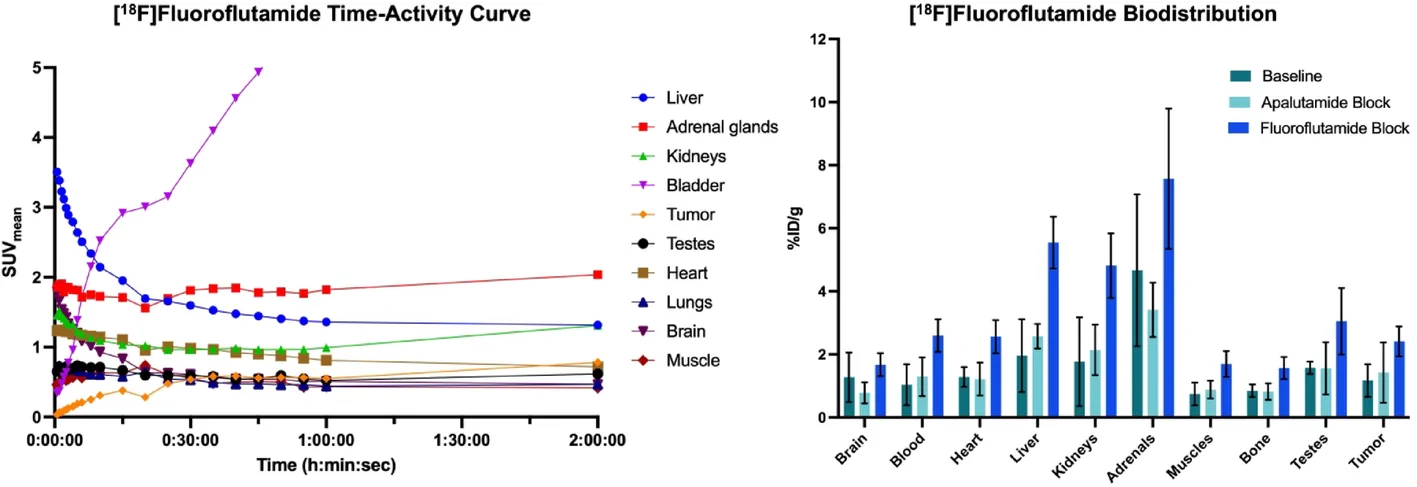
*Left*: Zoomed-in time-activity curve of [^18^F]fluoroflutamide uptake in the organs and tissues of a baseline mouse with a 22Rv1 xenograft. Bladder maxed out at an SUV_mean_ of 16.8 at 2 h. *Right*: Biodistribution of [^18^F]fluoroflutamide in 22Rv1 xenograft models shown as a percentage of injected dose per gram of tissue (%ID/g). Blocking doses were premixed with the tracer for a tail vein injection (apalutamide block *n* = 3; cold fluoroflutamide block *n* = 3). Baseline received the tracer exclusively via tail vein injection (*n* = 3).

In the time-activity curve for tissue uptake in the baseline mouse over time (0–2 h, Fig. 5), putative AR-expressing tissues, including 22Rv1 tumors and testes, display similar uptake values to muscle, which is commonly considered ‘background.’ The lack of uptake in the tumor and testes indicates nonspecific uptake rather than AR binding. This is also supported by the 2 h biodistribution data shown in Fig. 5. Across all treatment groups, the highest uptake was observed in the adrenal glands (baseline: 4.67% ID/g; apalutamide: 3.41% ID/g; cold fluoroflutamide: 7.57% ID/g), liver (1.96, 2.57, and 5.54% ID/g), and kidneys (1.77, 2.14, and 4.82% ID/g, respectively, Fig. 5). The tracer also exhibited minimal defluorination in vivo, as indicated by no increase of ^18^F uptake in bone (baseline average: 0.85% ID/g) compared to other background organs such as muscle (baseline average: 0.74% ID/g). Imaging data were consistent with biodistribution findings, with the bladder, adrenal glands, and liver exhibiting the highest SUVs at all time points and a modest increase in uptake observed with increasing cold mass.

Classical blocking effects were limited and only observed in the brain, heart, and adrenal glands in the apalutamide-treated cohort. Differences in adrenal uptake were not statistically significant (*P* > 0.05), and no classical blocking effects were observed in mice co-administered with cold fluoroflutamide. Instead, systemic uptake increased in both blocking groups relative to baseline. In both carrier-added groups, uptake increases in most organs at the 2 h time-point. This has previously been described as ‘mass action’ or ‘mass effect’(Kung and Kung, 2005). The addition of non-radioactive carrier or apalutamide, an analogous ligand, altered the pharmacokinetics and biodistribution over time by increasing tracer uptake in most organs, including the tumor. Since apalutamide and cold fluoroflutamide were co-formulated in polysorbate 80/EtOH/saline, formulation-dependent pharmacokinetic effects may also have contributed to the observed systemic increases in uptake. Changes in pharmacokinetics were observed when comparing radioactivity concentrations in the testes over time across groups (Fig. S24). The baseline mouse had an average SUV_mean_ of 0.64 ± 0.068 across all timepoints, while the carrier-added groups, apalutamide block, and cold fluoroflutamide block had initial uptake values of 0.22 and 0.24, respectively, at 30 seconds, which increased over time to 0.93 and 0.75 at 2 h. Therefore, another possible explanation for this observation is that adding mass alters pharmacokinetics and tissue distribution, leading to increased tracer retention across multiple tissues.

Some limitations of this study include the biodistribution study being conducted at a single time point (2 h), which may be later than desired for an AR ligand. The results of this initial study did not warrant generating more tumor models to assess biodistribution at earlier time points. Additionally, the binding affinity of the new fluorinated analog was not tested in vitro compared with that of flutamide and analogous antagonists. Further work involves in vitro binding assays with 22Rv1 cells and other AR-positive or AR-negative PC cell lines, such as PC-3 (AR^−^), DU145 (AR^−^), or LNCaP (AR^+^), and LAPC-4 (AR^+^) (Sampson et al. 2013).However, the biodistribution and imaging data suggest that the α-tertiary C–^18^F bond may suppress conversion to the higher-affinity 2-hydroxyflutamide metabolite. The lack of selective uptake is also consistent with reduced target engagement relative to 2-hydroxyflutamide, which has a 23-fold higher affinity to the AR than flutamide and accounts for 90% of metabolites present in plasma following a therapeutic dose (Simard et al. 1986). 

During the preparation of this manuscript, the Lee group published a new methodology for (Liu et al. 2018) ^18^F-trifluoromethylation of aryl iodides (Choi et al. 2025). Within this study, the group radiolabeled flutamide at the trifluoromethyl group with low molar activity, leaving the amide susceptible to α-hydroxylation via CYP1A2. When the agent was tested in LNCaP (AR^+^) xenograft mouse models, rapid renal clearance was observed, along with specific uptake in the xenograft up to 60 min post-injection. This further supports the role of α-C–H metabolism of flutamide in promoting AR targeting and suggests that the low uptake of **1** arises from reduced AR-binding avidity.

## Conclusions

We successfully developed an automated radiosynthesis of [^18^F]fluoroflutamide with high radiochemical purity and sufficient molar activity for preclinical imaging studies. In vivo studies in 22Rv1 xenograft-bearing mice demonstrated non-specific tumor uptake and minimal defluorination, as evidenced by the absence of significant skeletal signal. However, classical blocking with nonradioactive apalutamide or cold fluoroflutamide did not reduce tumor or tissue uptake. Instead, a paradoxical “mass effect” was observed, with increased tracer accumulation across multiple organs, including the tumor, when non-radioactive competitors were co-injected. These findings suggest that the in vivo distribution of [^18^F]fluoroflutamide is likely influenced by nonspecific pharmacokinetics and metabolism, rather than by AR-mediated binding. Overall, while [^18^F]fluoroflutamide is (bio)chemically stable and can be reliably synthesized, the current data indicate limited target engagement and specificity for AR imaging. Further optimization using structural analogs is required, particularly through in vitro binding assays against AR⁺ and AR⁻ cell lines, metabolite characterization, and evaluation in additional tumor models. For example, design strategies that preserve metabolically activated binding motifs may be necessary to achieve effective AR targeting. These studies and others are underway to identify effective, noninvasive tools for imaging AR expression in advanced PC, including CRPC and NEPC.

## Supplementary Information

Supplementary Material 1

## Data Availability

All data generated during this study, including chemistry/animal experiments, are included in this published article and its supplementary information files.

## Abbreviations

AR: Androgen receptor
EOB: End-of-Beam
mCRPC: Metastatic Castration-Resistant Prostate Cancer
PET: Positron Emission Tomography
PC: Prostate Cancer
CRPC: Castration-Resistant Prostate Cancer
NEPC: Neuroendocrine Prostate Cancer
PSMA: Prostate-Specific Membrane Antigen
[^18^F]FDHT: [^18^F]16β-fluoro-5α-dihydrotestosterone
[^18^F]FDG: [^18^F]Fluorodeoxyglucose
radio-HPLC: Radio-High-Performance Liquid Chromatography
NMR: Nuclear Magnetic Resonance
GE: General Electric
SPE: Solid Phase Extraction
TMAF: Tetramethylammonium fluoride
TMAOTf: Tetramethylammonium triflate
K_2.2.2_: Kryptofix
Zn(OTf)_2_: Zinc Triflate
TBD: Triazabicyclodecene
IACUC: Institutional Animal Care & Use Committee
MAP: Maximum a posteriori
KOTf: Potassium Triflate
DMSO: Dimethyl Sulfoxide
A_m_: Molar Activity
SUV: Standardized uptake value
RCY: Radiochemical yield
RCP: Radiochemical purity
