# Supplementary material for: Automated radiosynthesis and biodistribution of [18F]fluoroflutamide
Source: EJNMMI Radiopharm Chem. 2026 Jul 23;11:75. doi: 10.1186/s41181-026-00453-z (PMC13677656; doi:10.1186/s41181-026-00453-z)
Supplement: Supplementary file 1 [file 41181_2026_453_MOESM1_ESM.docx]

**sAutomated Radiosynthesis and Biodistribution of [^18^F]Fluoroflutamide**

Gina Kaup,^1^ Jason A. Witek,^1^ Shelbie Cingoranelli,^1^ Jenelle Stauff,^1^ David Raffel,^1^ Allen F. Brooks,^1^ Peter J. H. Scott,^1,3,*^ Jay S Wright^2*^

*^1^* *Division of Nuclear Medicine, Department of Radiology, University of Michigan Medical School, Ann Arbor, MI 48109, United States, ^2^* *Department of Radiology, Perelman School of Medicine, University of Pennsylvania, Philadelphia, PA 19104, United States, ^3^The Interdepartmental Program in Medicinal Chemistry, University of Michigan, Ann Arbor, MI 48109, United States, *Correspondence: JSW (email: jswright@upenn.edu), PJHS (email: pjhscott@med.umich.edu)*

Table of Contents

1. Chemistry and Characterization	2

1.1 General Considerations	2

1.2. Synthetic Procedures	2

- Synthesis of Bromide Precursor	2
- Synthesis of Fluorine Standard	3

2. Radiochemistry	3

2.1. General Considerations	3

2.2 General Procedure for Radiochemical Synthesis	5

2.3 Radiochemical Syntheses Data and HPLC Traces	7

3. Animal Studies	15

3.1. Supplemental Data from PET Imaging Studies	15

4. NMR Data	17

5. References	20

**1. Chemistry and Characterization**

**1.1. General Considerations**

All chemicals were purchased from commercial suppliers and used without purification. Automated flash chromatography was performed with a Biotage Isolera Prime system. High-performance liquid chromatography (HPLC) was performed using a Shimadzu LC-2010A HT system equipped with a Bioscan B-FC-1000 radiation detector. ^1^H and ^13^C NMR spectra: Varian apparatus (400 MHz for ^1^H NMR and 100 MHz for ^13^C NMR), in CDCl_3_ unless otherwise indicated, δ in ppm relative to tetramethylsilane (δ = 0), J in Hz. Mass spectrometry (HRMS) was performed on an Agilent 6520 Accurate-Mass Q-TOF LC/MS spectrometer using ESI ionization, with less than 5 ppm error for all HRMS analyses. 2-Bromo-2-methylpropanoyl bromide, triethylamine, anhydrous bottles of acetonitrile (MeCN, ≥99.9%) and dichloromethane (DCM, 99.9%) fitted with an AcroSeal™ septum, and silver (II) fluoride were purchased from Thermo Fisher Scientific. 3-Nitro-3-(trifluoromethyl)aniline was purchased from Oakwood Chemical. Deuterated chloroform (CDCl_3_) for NMR analysis was purchased from Cambridge Isotope Laboratories. Compounds **1** and **2** were previously reported by Mizuta, and the reported methods were followed with slight variation.^1^

**1.2. Synthetic Procedures**

***Synthesis of Bromide Precursor***

**2-Bromo-2-methyl-N-(4-nitro-3-(trifluoromethyl)phenyl)propanamide 2.** Triethylamine (1.00 mL, 7.20 mmol) and 2-bromo-2-methylpropanoyl bromide (758.55 µL, 6.14 mmol) were added to a solution of 4-nitro-3-(trifluoromethyl)aniline (1.10 g, 5.34 mmol) and dichloromethane (15 mL) at 0°C and allowed to stir at room temperature for 23 hours. The reaction mixture was washed with saturated NaHCO_3_ (2 x 15 mL), brine (1 x 20 mL), and dried over anhydrous Na_2_SO_4_. The resulting organic layers were concentrated *in vacuo* and purified using flash column chromatography (0-50% EtOAc/HEX) to afford 2-bromo-2-methyl-*N*-(4-nitro-3-(trifluoromethyl)phenyl)propenamide as a white crystalline powder (1.11g, 58.39% yield). Data for **1** matched a report by Mizuta.^1^

δ^1^H (599 MHz, CDCl_3_) 8.77 (br s, 1H), 8.05 (d, J = 2.4 Hz, 1H), 8.02-7.95 (m, 2H), 2.06 (s, 6H).

δ^13^C (151 MHz, CDCl_3_) 170.8, 143.7, 141.7, 127.2, 125.5 (q, J = 34 Hz), 122.7, 121.8 (q, J = 275 Hz), 118.6 (q, J = 6 Hz), 62.1, 32.4.

**Synthesis of Fluoroflutamide Standard**

**2-Fluoro-2-methyl-N-(4-nitro-3-(trifluoromethyl)phenyl)propanamide 1-^19^F.** A 20 mL scintillation vial equipped with a magnetic stir bar was charged with compound **2** (2-bromo-2-methyl-N-(4-nitro-3-(trifluoromethyl)phenyl)propenamide (44 mg, 0.15 mmol), AgF (38 mg, 0.3 mmol), and MeCN (0.1M) and allowed to stir at room temperature for 12 h. The resulting mixture was diluted with EtOAc and filtered through a celite plug. The plug was then washed with EtOAc (10 mL), and the filtrate was concentrated in vacuo. Purification via flash column chromatography in an EtOAc/hexanes system afforded the desired compound as an off-white solid (32 mg, 71% yield). Data for **1-^19^F** matched a report by Mizuta.^1^

δ^1^H (599 MHz, CDCl_3_) 8.46 (br d, *J* = 9.0 Hz, 1H), 8.09 (s, 1H), 8.05-7.97 (m, 2H), 1.69 (d, *J* = 18 Hz, 6H).

δ^13^C (151 MHz, CDCl_3_) 172.0 (d, *J* = 21 Hz), 143.7, 141.3, 127.2, 125.6 (q, *J* = 34 Hz), 124.5, 121.8 (q, *J* = 275 Hz) 118.7 (q, *J* = 6 Hz), 96.7 (d, *J* = 181 Hz), 25.1 (d, *J* = 24 Hz).

δ^19^F (563 MHz, CDCl_3_) -60.13 (s, 3F), -145.48 (m, 1F).

**2. Radiochemistry**

**2.1. General Considerations**

Reagents and solvents were commercially available unless otherwise stated. QMA-light (WAT023525), Alumina N Plus Light (WAT023561), and Oasis HLB 1 cc Vac (WAT094225) cartridges were purchased from Waters Corporation. QMA-light Sep-Paks were flushed with 10 mL of ethanol, 10 mL of aqueous 0.5 M KOTf, and 10 mL of Milli-Q^®^ water before use. Alumina N Plus Light Sep-Pak cartridges and Oasis HLB 1 cc Vac cartridges were flushed with 10 mL of ethanol and 10 mL of water before use. Triazabicyclodecene, flutamide, apalutamide, Millex™-FG sterile filters, Millex™-GV sterile filters, and anhydrous bottles of dimethylsulfoxide (DMSO, ≥99.9%) fitted with a Sure/SealTM septum were purchased from Millipore Sigma. Zinc triflate and anhydrous bottles of acetonitrile (MeCN, ≥99.9%) fitted with an AcroSeal™ septum were purchased from ThermoFisher Scientific. Sodium chloride, 0.9% USP and sterile water for injection, USP were purchased from Pfizer. ASCOR® ascorbic acid injection (500 mg/mL), USP was purchased from McGuff Pharmaceuticals. Sterile dose vials were purchased from ALK Life Science Solutions™.

Tetramethylammonium triflate (Me_4_NOTf) was synthesized by the slow addition of Me_4_NOH pentahydrate (10 g, 55.2 mmol, 1 equiv.) in 20 mL H_2_O to a 0 °C solution of Tf_2_O (4.6 mL, 27.6 mmol, 0.5 equiv.), in H_2_O (20 mL), over 20 min. The reaction was allowed to warm to room temperature over 1 h before azeotropic removal of the volatiles with MeCN (3 × 75 mL) and recrystallization from anhydrous isopropanol (iPrOH) to obtain a solid residue that was dried under high vacuum (0.1 Pa) at 150 °C for 3 d. Me_4_NOTf was obtained as a free-flowing white powder (11.1 g, 49.7 mmol, 90%), with characterization data matching previous reports.^2–4^ Radiochemical yields reported as non-decay corrected.

**2.2. General Procedure for Radiochemical Syntheses**

The GE TRACERlab FX_N-Pro_ in standard configuration was loaded as follows: Sep-Pak^TM^ Plus Light QMA prepped with 10 mL of EtOH, 10 mL of 0.5 M KOTf, 10 mL of Milli-Q water, Sep-Pak^TM^ Plus Light Alumina N cartridge prepped with 10 mL EtOH, 10 mL of Milli-Q water, Waters Oasis® HLB 1 cc/30 mg prepped with 10 mL EtOH, 10 mL of Milli-Q water, Vial #1: TMAOTf (5.9 mg, 26.5 µmol) in 0.5 mL H_2_O, Vial #2: K_2.2.2_ in 1 mL MeCN (0.14 mg, 0.36 µmol), Vial #3:	Zn(OTf)_2_ (20 mol%, 0.36 mg) and triazabicyclodecene (TBD, 0.9 equiv., 0.63 mg) in 400 µL MeCN + 100 µL MeCN, Vial #4: Precursor (1 equiv., 1.8 mg, 5 µmol) in 500 µL MeCN, Vial #5: 3.5 mL semi-prep HPLC eluent, Vial #12: 4.5 mL of USP saline, Vial #13: 500 µL USP EtOH, Vial #14: 10 mL MQ H_2_O, Product vial: 5.0 mL USP saline, 0.1 mL USP ascorbic acid solution, Dilution flask: 60 mL H_2_O, 0.1 mL USP ascorbic acid solution.

*Figure S1: Standard configuration of GE TRACERlab FX_N-Pro_.*

*Figure S2: Time list for the production of [^18^F]fluoroflutamide using a GE TRACERlab FX_N-Pro_.*

**2.3. Radiochemical Syntheses Data and HPLC Traces**

| **Run 1** | |
| --- | --- |
| Activity Yield | 81.0 mCi |
| ^18^F Activity at EOB | 1.87 Ci |
| Radiochemical Yield | 4.34% |
| Radiochemical Purity | 99.9% |
| Molar Activity | 2,336.43 Ci/mmol |
| 2 h Stability RCP | 45.25% |

*Figure S3: Analytical HPLC traces of [^18^F]fluoroflutamide dose from run 1.*

*Figure S4: Analytical HPLC trace of ^19^F-fluoroflutamide standard from run 1.*

*Figure S6: Semi-preparative HPLC traces of the automated synthesis of [^18^F]fluoroflutamide from run 1.*

*Figure S5: Analytical HPLC traces of [^18^F]fluoroflutamide dose from run 1 coinjected with ^19^F-fluoroflutamide standard.*

*Figure S7: Standard curve for the molar activity calculation from run 1.*

*Figure S8: Analytical HPLC traces of [^18^F]fluoroflutamide dose from run 1 coinjected with flutamide at 2 h post EOS. Radiolysis observed.*

Run 1 Standard Curve

| Concentration (mg/mL) | Area Under the Curve |
| --- | --- |
| 0.01 | 55656 |
| 0.005 | 26847 |
| 0.001 | 5372 |
| 0.0005 | 2556 |
| 0.0001 | 447 |

| **Run 2** | |
| --- | --- |
| Activity Yield | 87.7 mCi |
| ^18^F Activity at EOB | 1.87 Ci |
| Radiochemical Yield | 4.70% |
| Radiochemical Purity | 99.9% |
| 1 h Stability RCP | 99.54% |
| 4 h Stability RCP | 97.80% |

*Figure S10: Analytical HPLC trace of ^19^F-fluoroflutamide standard from run 2.*

*Figure S9: Analytical HPLC traces of [^18^F]fluoroflutamide* *dose from run 2.*

*Figure S13: Semi-preparative HPLC traces of the automated synthesis of [^18^F]fluoroflutamide from run 2.*

*Figure S12: Analytical HPLC trace of [^18^F]fluoroflutamide* *dose 4-hours post-EOS from run 2.*

*Figure S11: Analytical HPLC trace of [^18^F]fluoroflutamide* *dose 1-hour post-EOS from run 2.*

*Figure S14: Analytical HPLC traces of [^18^F]fluoroflutamide* *dose from run 3*

| **Run 3** | |
| --- | --- |
| Activity Yield | 54.2 mCi |
| ^18^F Activity at EOB | 1.87 Ci |
| Radiochemical Yield | 2.90% |
| Radiochemical Purity | 99.81% |

*Figure S15: Analytical HPLC traces of [^18^F]fluoroflutamide dose from run 3 coinjected with ^19^F-fluoroflutamide standard.*

*.*

*Figure S16: Analytical HPLC trace of ^19^F-fluoroflutamide standard from run 3*

*Figure S17: Semi-preparative HPLC traces of the automated synthesis of [^18^F]fluoroflutamide from run 3.*

| **Run 4** | |
| --- | --- |
| Activity Yield | 35.3 mCi |
| ^18^F Activity at EOB | 1.87 Ci |
| Radiochemical Yield | 1.89% |
| Radiochemical Purity | 99.9% |
| Molar Activity | 811.34 Ci/mmol |

*Figure S18: Analytical HPLC trace of [^18^F]fluoroflutamide dose from run 4.*

*Figure S20: Analytical HPLC traces of [^18^F]fluoroflutamide dose from run 4 coinjected with ^19^F-fluoroflutamide standard.*

*Figure S21: Semi-preparative HPLC traces of the automated synthesis of [^18^F]fluoroflutamide from run 4.*

*Figure S19: Analytical HPLC trace of ^19^F-fluoroflutamide standard from run 4.*

*Figure S22: Standard curve for the molar activity calculation from run 4.*

Run 4 Standard Curve

| Concentration (mg/mL) | Area Under the Curve |
| --- | --- |
| 0.01 | 45447 |
| 0.005 | 22450 |
| 0.001 | 4420 |
| 0.0005 | 2187 |
| 0.0001 | 356 |

**3. Animal Studies**

**3.1 Supplemental Data from PET Imaging Studies**

*Figure S23: Full time-activity curves of [^18^F]fluoroflutamide uptake in a baseline mouse and carrier-added groups (apalutamide block and ^19^F-fluoroflutamide block) with 22Rv1 xenografts. The top three graphs are zoomed in while the bottom three graphs include the bladder in the curve.*

*Figure S24: Time-activity curves of [^18^F]fluoroflutamide uptake in the tumor and testes of a baseline mouse and carrier-added groups with 22Rv1 xenografts.*

**4. NMR Spectra**

**5. References**

(1)	Mizuta, S.; Kitamura, K.; Kitagawa, A.; Yamaguchi, T.; Ishikawa, T. Silver-Promoted Fluorination Reactions of α-Bromoamides. *Chem. Eur. J.* **2021**, *27* (19), 5930–5935. https://doi.org/10.1002/chem.202004769.

(2)	Wright, J. S.; Ma, R.; Webb, E. W.; Winton, W. P.; Stauff, J.; Cheng, K.; Brooks, A. F.; Sanford, M. S.; Scott, P. J. H. Zinc-Mediated Radiosynthesis of Unprotected Fluorine-18 Labelled α-Tertiary Amides. *Angew. Chem. Int. Ed.* **2024**, *63* (2), e202316365. https://doi.org/10.1002/anie.202316365.

(3)	Sagl, D. J.; Martin, J. C. The Stable Singlet Ground State Dication of Hexaiodobenzene: Possibly a .Sigma.-Delocalized Dication. *J. Am. Chem. Soc.* **1988**, *110* (17), 5827–5833. https://doi.org/10.1021/ja00225a038.

(4)	Toro, J. M. S.; Hartog, T. den; Chen, P. Cyclopropanation of Styrenes and Stilbenes Using Lithiomethyl Trimethylammonium Triflate as Methylene Donor. *Chem. Commun.* **2014**, *50* (73), 10608–10610. https://doi.org/10.1039/C4CC04929B.
